# Biological Potential of Sixteen Legumes in China

**DOI:** 10.3390/ijms12107048

**Published:** 2011-10-20

**Authors:** Yang Yao, Xuzhen Cheng, Lixia Wang, Suhua Wang, Guixing Ren

**Affiliations:** Institute of Crop Science, Chinese Academy of Agricultural Sciences, South Xueyuan Road, Haidian District No.80, Beijing 100081, China; E-Mails: yaoyang@caas.net.cn (Y.Y.); chengxz@caas.net.cn (X.C.); wanglx@caas.net.cn (L.W.); wangsh@caas.net.cn (S.W.)

**Keywords:** legumes, antioxidant, α-glucosidase inhibition, advanced glycation endproducts, tyrosinase inhibition

## Abstract

Phenolic acids have been identified in a variety of legumes including lima bean, broad bean, common bean, pea, jack bean, goa bean, adzuki bean, hyacinth bean, chicking vetch, garbanzo bean, dral, cow bean, rice bean, mung bean and soybean. The present study was carried out with the following aims: (1) to identify and quantify the individual phenolic acid and determine the total phenolic content (TPC); (2) to assess their antioxidant activity, inhibition activities of α-glucosidase, tyrosinase, and formation of advanced glycation endproducts; and (3) to investigate correlations among the phytochemicals and biological activity. Common bean possesses the highest antioxidant activity and advanced glycation endproducts formation inhibition activity. Adzuki bean has the highest α-glucosidase inhibition activity, and mung bean has the highest tyrosinase inhibition activity. There are significant differences in phytochemical content and functional activities among the bean species investigated. Selecting beans can help treat diseases such as dermatological hyperpigmentation illness, type 2 diabetes and associated cardiovascular diseases.

## 1. Introduction

Legumes as a functional food ingredient have gained a lot of interest [1,2]. Proteins [3], saponins [4] and phenolic compounds [5] present in beans are active ingredients responsible for benefits associated with the consumption of beans. Phenolic compounds have been reported to reduce the risk of cancer, heart disease, and diabetes, as well as have antibacterial, antiviral, anti-inflammatory, and anti-allergenic activities. Many of these benefits result from the antioxidant characteristics [6]. Antioxidants refer to compounds possessing free radical-scavenging activity, transition metal-chelating activity, and/or singlet oxygen-quenching capacity [7,8]. Several studies have suggested that the cells of diabetic patients are under oxidative stress with an imbalance between free radical-generating and radical-scavenging capacities. The increased free radical production and reduced antioxidant defense may partially mediate the initiation and progression of diabetes-associated complications [9,10].

Acting as a key enzyme for carbohydrate digestion, intestinal α-glucosidase is one of the glucosidases located at the epithelium of the small intestine. α-Glucosidase has been recognized as a therapeutic target for modulation of postprandial hyperglycaemia, which is the earliest metabolic abnormality to occur in type 2 diabetes mellitus [11]. Inhibition of intestinal α-glucosidases delays the digestion and absorption of carbohydrates, thereby suppressing postprandial hyperglycaemia [12]. Advanced glycation end products (AGEs) are part of a major pathogenic process in diabetic complications including neuropathy, nephropathy, retinopathy, atherosclerosis and cataracts [13]. Thus, the discovery and investigation of AGE inhibitors would offer a potential therapeutic approach for the prevention of diabetic complications.

Free radicals can up-regulate the mRNA level for tyrosinase, and amplify the production of melanin that correlates directly with serious problems in human skin [14]. Acting as a key enzyme for synthesis of melanin pigments, tyrosinase catalyzes two distinct reactions in melanin synthesis: the hydroxylation of l-tyrosine to l-dopa and the oxidation of l-dopa to dopaquinone, after further series of conversions to the melanin produced [15]. The inhibitors of tyrosinase have been used to treat some dermatological hyperpigmentation illness connected with overproduction of melanin, which also play a significant role in the cosmetic business as a skin whitening agent [16].

Phenolic acids have been reported in some legumes, however, there is still a lack of systematic study of diverse species beans on the relative abundance of total phenolic content (TPC), and their antioxidant, α-glucosidase inhibition, advanced glycation endproducts formation inhibition, and tyrosinase inhibition activities.

## 2. Materials and Methods

### 2.1. Materials

Fifteen edible beans were grown in China in 2009. The names and places of production of the beans are shown in Table 1. Standards of *p*-hydroxybenzoic acid, gentisic acid, vanillic acid, caffeic acid, chlorogenic acid, syringic acid, *p*-coumaric acid, ferulic acid, sinapic acid, *m*-coumaric acid, gallic acid, Trolox, 1,1-diphenyl-2-picrylhydrazyl radical (DPPH), Folin-Ciocalteu phenolic reagent, rat intestinal acetone powder, bovine serum albumin(BSA), d-glucose, methylglyoxal (MGO), l-DOPA and mushroom tyrosinase were purchased from Sigma-Aldrich (St. Louis, MO, USA). All of the chemicals were of analytical grade and were obtained from Beijing Chemical Reagent (Beijing, China). All of the analytical grade solvents for high performance liquid chromatography (HPLC) were purchased from Fisher Chemicals (Shanghai, China).

### 2.2. Extraction

All dried samples were ground in a laboratory mill and passed through a sieve (80 mesh). Bean samples (10 g) were extracted twice in 100 mL of 70% ethanol for 2 h at room temperature. After vacuum filtration, the supernatants were combined and concentrated under reduced pressure in a rotary evaporator at 50 °C. After freeze-drying, the sample powder was stored at −20 °C until analysis. The biological activities of the azuki beans were measured at a concentration of 15 mg/mL.

### 2.3. HPLC Analysis of Individual Phenolic Acids

HPLC system was equipped with two Shimadzu LC-20A pumps, a Shimadzu LC-20 autosampler, a SPD-20A UV/vis detector and an Alltima C18 column (4.6 mm × 250 mm, Metachem Technologies Inc., Torrance, CA). The wavelength of the UV detector was set at 280 nm. The mobile phase was a mixture of solvent A (HPLC water containing 0.05% TFA) and solvent B (acetonitrile: MeOH: TFA = 30:10:0.05). The gradient elution was programmed as follows: from 10% to 12% B in 16 min; from 12% to 25% B in 9 min; from 25% to 50% B in 25 min; from 50% to 75% B in 18 min; from 75% to 10% B in 10 min. The flow rate was set at 1.0 mL/min, and the injection volume was 10 μL. Each phenolic acid was quantified according to its calibration curve.

### 2.4. Determination of Total Phenolic Content (TPC)

TPC was measured using the Folin-Ciocalteu method described previously [17,18]. μBriefly, 50 L of the extract was mixed in 5 mL of distilled deionised water followed by the addition of 500 μL of 1 M Folin-Ciocalteu reagent and 500 μL of a 20% (w/v) Na_2_CO_3_ solution. The mixture was thoroughly mixed and allowed to stand for 60 min at room temperature before the absorbance was measured at 765 nm (Bio-Rad Smart Spec Plus Spectrophotometer, Hercules, USA). Quantification was performed with respect to the standard curve of gallic acid. The results were expressed as milligrams of gallic acid equivalent (GAE) per gram.

### 2.5. Antiradical Activity Against DPPH Ridical

The DPPH radical-scavenging activity was determined using the method reported by Yen and Chen [17]. DPPH (100 μM) was dissolved in 96% ethanol. The DPPH solution (1 mL) was mixed with 1 mL of the extract solution. The mixture was shaken and allowed to stand at room temperature in the dark for 10 min. The decrease in absorbance of the resulting solution was measured at 517 nm after 10 min. The results were expressed in micromoles of Trolox equivalents (TE) per gram.

### 2.6. Determination of α-Glucosidase Inhibition Activity

The α-glucosidase inhibition activity was determined as described previously [19]. α-Glucosidase (1 U/mL) inhibition activity was assayed using 50 μL of extracts with varying concentrations incubated with 100 μL of 0.1 M phosphate buffer (pH 7.0) in 96-well plates at 37 °C for 10 min. After preincubation, 50 μL of 5 mM p-nitrophenyl-α-d-glucopyranoside in a 0.1 M phosphate buffer (pH 7.0) was added to each well. The reaction mixtures were incubated at 37 °C for 5 min. The absorbance readings were recorded at 490 nm on a microplate reader before and after incubation (BioRad, IMAX, Hercules, USA). The results were expressed as a percent of α-glucosidase inhibition, and the inhibition activity was calculated according to the following equation: (A_control_ – A_sample_)/A_control_ × 100%.

### 2.7. Evaluation of AGE Inhibition Activity

BSA-glucose and BSA-MGO models were used for the evaluation of the inhibition effect of the extracts on the formation of advanced glycation end products. The BSA-glucose assay was carried out according the method reported by Peng and others [13]. Briefly, 5 g of BSA and 14.4 g of d-glucose were dissolved in 1.5 M phosphate buffer (pH 7.4) to obtain the control solution with 50 mg/mL BSA and 0.8 M d-glucose. Two milliliters of the control solution was incubated at 37 °C for 7 days in the presence or absence of 1 mL of bean extracts in a 1.5 M phosphate buffer (pH 7.4). After 7 days of incubation, fluorescent intensity (excitation at 330 nm and emission at 410 nm) was measured. Percent inhibition of AGE formation by each extract was calculated using the following equation: (1-(fluorescence of the solution with inhibitors/fluorescence of the solution without inhibitors)) × 100%.

The BSA-MGO assay was carried out according to the method reported by Yao and others [20]. Briefly, 40 mg of BSA was mixed with 31 μL of MGO in a 0.1 M phosphate buffer (pH 7.4) to obtain the control solution with 1 mg/mL BSA and 5 mM MGO. Two milliliters of the control solution was incubated at 37 °C for 6 days with or without 1 mL of the bean extracts in phosphate buffer. The percent inhibition was calculated based on the equation applied in the BSA-glucose assay as described above (excitation at 340 nm and emission at 420 nm).

### 2.8. Measurement of Tyrosinase Inhibition Activity

The tyrosinase activity was determined as described previously [21]. Assays were conducted in a 96-well microtiter plate and a plate reader was used to measure absorbance at 475 nm. Each well contained 40 μL of sample with 80 μL of phosphate buffer (0.1 M, pH 6.8), 40 μL of tyrosinase (31 units/mL) and 40 μL of l-DOPA (2.5 mM), the samples were incubated for 30 min at 37 °C. Control had all the components except tyrosinase. The percentage tyrosinase inhibition was calculated as follows: (A_control_ – A_sample_)/A_control_ × 100%

### 2.9. Statistical Analysis

All values were expressed as mean ± SD. Data were analyzed using one-way analysis of variance (ANOVA) followed by Tukey-Kramer test (Matlab version 7.6).

## 3. Results and Discussion

### 3.1. Individual Phenolic Acid, Total Phenolic Content (TPC) and Antioxidant Activity

Five phenolic acids (caffeic acid, chlorogenic acid, *p*-coumaric acid, ferulic acid and sinapic acid) were found in those beans, the contents of individual phenolic acid in the different bean samples are shown in Table 2. It was found that ferulic acid was the dominant phenolic acid in all beans, the highest content was 26.06 ± 2.19 mg/100 g in common bean and the lowest one was 9.10 ± 1.29 mg/100 g in garbanzo bean. The highest *p*-coumaric acid was found in common bean, 10.3 times higher than that in goa bean, which was the lowest among all the bean samples investigated in this study.

It was also found that TPC as measured by Folin-Ciocalteu method varied widely in legumes. Phenolic compounds are considered as the major compounds that contribute to the total antioxidant activities of the grains [19]. In the present study, common bean, with an average of 8.59 ± 0.11 mg GAE/g, was found to possess the highest TPC among all of the studied legumes and had 8.3 times greater than that of garbanzo bean (1.04 ± 0.24 mg GAE/g). Mung bean (8.14 mg GAE/g) had a high level of phenolics, this observation is in agreement with that of Peng *et al* [13]; they found that mung bean extract had the highest TPC among mung bean, black bean, soybean and cow bean.

The antioxidant activities of legume extracts were evaluated by measuring their DPPH radical scavenging activities. All of the extracts exhibited strong antioxidant activities (Table 3), DPPH showed the same trends as did in TPC. Among the tested samples, common bean had the highest DPPH free radical scavenging activity (46.83 μM TE/g), whereas garbanzo bean had the lowest DPPH free radical scavenging activity (1.28 μM TE/g). Our results on DPPH in soybean (15.17 μM TE/g) were in agreement with that (18.44 μM TE/g) reported previously [22], the results from garbanzo bean (1.28 μM TE/g) were in agreement with that (1.05–1.24 μM TE/g) of the previous report [23], while the other results from pea (31.92 μM TE/g), and common bean (46.83 μM were higher than respective values (2.25, 18.95 μM TE/g) in a previous report based on dry weight [22]. Differences between our results and previous reports may be attributed partly to the differences in the sources of materials and in expressions based on dry weight or fresh basis calculation. It is difficult to compare our data to that reported by Amarowicz and Ronaldsince [24] as they were expressed in a different unit.

It was found that TPC were highly correlated with their antioxidant activity (*p* < 0.01). Similar effect was found in the study by Yao *et al*. [19] who investigated seven color grains and found the antioxidant activity showed a positive correlation with their TPC. Antioxidant activity of phenolics depends on the structure and substitution pattern of hydroxyl groups. *p-*Coumaric can exhibit competitive antioxidant activity because of the 4-position of hydroxylation on the phenolic ring and the additional conjugation in the propenoic side chain, which might facilitate the electron delocalization, by resonance, between the aromatic ring and propenoic group gives high antioxidant activity [25].

### 3.2. α-Glucosidase Inhibition Activities

Table 3 shows that adzuki bean had the highest α-glucosidase inhibition activity (64.33%), followed by the Goa bean (60.42%). Itoh *et al.* [26] investigated the antidiabetic effects of azuki beans on streptozotocin (STZ)-induced diabetic rats, and they suggested that the active fraction of azuki beans suppresses the postprandial blood glucose by inhibiting α-glucosidase. The inhibition in common bean, cow bean and rice bean was higher than 50%. α-Glucosidase inhibition was not statistically correlated with their phenolic acids and antioxidant activities of the extracts (Table 4). Mccue and others [27] investigated fifteen Asian beans, fruits and vegetables, and they concluded that a high phenolic content does not always confer a high inhibition of α-glucosidase activity of a food extract, which may be due to the nonphenolic compounds in the samples.

### 3.3. Advanced Glycation Endproducts Formation Inhibition Activities

The inhibition measured by BSA-glucose varied significantly among different beans (Table 3). Common bean had the highest inhibition (86.67%), followed by mung bean (74.84%). The inhibition measured by BSA-MGO showed the same trends as did BSA-glucose method. Common bean exhibited the highest inhibition (74.06%), followed by mung bean (72.67%). Beans have been recommended as suitable foods for diabetic patients in the past mainly based on their high fiber and protein contents [28]. Recently, it has been reported that beans contained considerable bioactive phytochemicals, including phenolic compounds, which offer extra benefits for amelioration of diabetes and alleviating diabetic complications [29]. The results (Table 4) obtained in our study showed that BSA-MGO and BSA-gluocose significantly correlate with TPC assay (*p* < 0.05). Similar results have been observed by Peng and others [12] who investigated the correlation of the total phenolic content and inhibition effect of the phenolics on the formation of advanced glycation end products of mung bean, black bean, soybean and cowpea, and they demonstrated that phenolic compounds inhibit the formation of advanced glycation end products by inhibition of free radical generation in the glycation process and subsequent inhibition of protein modifications.

### 3.4. Tyrosinase Inhibition Activities

In present study, mung bean with an average inhibition of 81.24%, had the highest tyrosinase inhibition activities among all the legumes and was 3.5 times higher than that of garbanzo bean (21.35%). Common bean (75.89%) also had a high level of inhibition. To our knowledge, this is the first report that edible beans have tyrosinase inhibition activities. The results showed that tyrosinase inhibition activity significantly correlates with TPC and DPPH assays (*p* < 0.01) (Table 4). Gomez-Cordoves and others [30] reported phenolic fractions inhibit melanogenic activity in melanocytes and decrease colony forming of melanoma cells, which support their potential as therapeutic agents in the treatments of human melanoma. Melanogenesis is activated by oxidation related processes such as UV radiation. Melanogenesis requires tyrosinase activity and reactive species such as reactive oxygen and nitrogen species cause oxidative stress to the skin resulting in skin pigmentation and ageing [31]. Hence, controlling oxidative stress is important for the regulation of melanogenesis, since the antioxidant may be closely related to anti-melanogenic actions and regulation of melanin synthesis [32]. This was recently confirmed by Abdillahi [33] and Wu [34], where the anti-melanogenic activity of both Podocarpus and Taiwanese were attributed to their antioxidative actions.

## 4. Conclusion

In summary, there are significant differences in phytochemical content and functional activities among the bean species investigated. Common bean appeared to possess the highest antioxidant activity, adzuki bean has the highest α-glucosidase inhibition and advanced glycation endproducts formation, and mung bean has the highest tyrosinase inhibition activity among all the beans tested. These results provide useful information when selecting bean species for better design of potential functional food that can treat diseases such as dermatological hyperpigmentation illness, type 2 diabetes and associated cardiovascular diseases.

## Figures and Tables

**Table 1:** The names and places of production of edible beans collected from China.

	Latin name	Cultivars	Place of production
Lima bean	*Phaseolus lunatus*	Yu-Shan-Bai-Yu-Dou	Jiangxi
Broad bean	*Vicia faba*	Feng-Dou No.1	Yunnan
Common bean	*Phaseolus vulgaris*	Long 2244	Heilongjiang
Pea	*Pisum sativum*	Ding-Wan No.1	Gansu
Jack bean	*Canavalia ensiformis*	Xian-Lv	Zhejiang
Goa bean	*Psophocarpus tetragonolobus*	Gui-Feng No.1	Guangxi
Adzuki bean	*Vigna angularis*	Jing-Xiao No.38	Beijing
Hyacinth bean	*Dolichos lablab*	Gan-Bian No.5	Gansu
Chicking vetch	*Lathyrus sativus*	Lon-Xian	Heilongjiang
Garbanzo bean	*Cicer arietium*	A-1	Xinjiang
Dral	*Cajanus cajan*	Gui-Mu No.2	Guangxi
Cow bean	*Vigna unguiculata*	Zao-Jiang No.1	Jiangsu
Rice bean	*Vigna umbellata*	Man-Dou	Sichuan
Mung bean	*Glycine max*	Zhong-Lv No.5	Shanxi
Soybean	*Phaseolus aureus*	Hua-Dou No.20	Henan

**Table 2:** Average concentration of phenolic acid in beans (in mg/100 g).

	Caffeic acid	Chlorogenic acid	*p*-Coumaric acid	Ferulic acid	Sinapic acid	Total
Lima bean	nd	nd	3.25 ± 0.68 ^f,g^	15.12 ± 2.15 ^f,g^	6.74 ± 0.15 ^c^	25.11 ± 1.07 ^d^
Broad bean	0.78 ± 0.03 ^c^	nd	1.68 ± 0.07 ^i,j,k^	10.56 ± 1.58 ^j^	2.58 ± 0.23 ^i,h^	15.60 ± 1.33 ^g^
Common ean	nd	0.18 ± 0.05 ^a^	11.10 ± 0.16 ^a^	26.06 ± 2.19 ^a^	9.55 ± 0.24 ^a^	45.89 ± 2.19 ^a^
Pea	0.53 ± 0.01 ^e^	nd	6.12 ± 1.14 ^d^	11.66 ± 1.93 ^i^	3.92 ± 0.19 ^e,f^	22.23 ± 1.65 ^e^
Jack bean	nd	nd	1.84 ± 0.31 ^j,i^	11.72 ± 2.10 ^h,i^	8.33 ± 0.61 ^a,b^	21.89 ± 1.60 ^e^
Goa bean	nd	nd	1.08 ± 0.27 ^j,k^	10.39 ± 2.01 ^j^	6.74 ± 0.38 ^c^	18.21 ± 1.94 ^f^
Adzuki bean	1.12 ± 0.15 ^a^	0.20 ± 0.07 ^a^	2.68 ± 0.51 ^g,h^	15.41 ± 1.36 ^f^	6.80 ± 0.26 ^c^	25.01 ± 0.76 ^d^
Hyacinth bean	0.96 ± 0.10 ^b^	nd	4.63 ± 0.88 ^e^	19.05 ± 2.75 ^c,d^	3.25 ± 0.16 ^f,g,h^	28.25 ± 1.75 ^c^
Chicking vetch	nd	nd	2.67 ± 0.19 ^g,h^	12.39 ± 1.63 ^h^	2.27 ± 0.30 ^l^	17.33 ± 1.99 ^g,f^
Garbanzo bean	nd	0.13 ± 0.01 ^b^	4.50 ± 0.53 ^e^	9.10 ± 1.29 ^k^	4.47 ± 0.41 ^e^	18.20 ± 0.87 ^f^
Dral	nd	nd	8.78 ± 0.67 ^b^	18.39 ± 2.01^d,e^	7.85 ± 0.62 ^b^	35.02 ± 1.63 ^b^
Cow bean	nd	nd	7.84 ± 0.25 ^c^	19.48 ± 2.30 ^b,c^	6.22 ± 0.51^c^	33.54 ± 1.96 ^b^
Rice bean	0.73 ± 0.02 ^c,d^	0.15 ± 0.02 ^b^	3.79 ± 0.16 ^f^	18.23 ± 1.49 ^e^	3.49 ± 0.33 ^f,g^	26.39 ± 1.20 ^c,d^
Mung bean	nd	nd	5.76 ± 0.22 ^d^	19.86 ± 1.25 ^b^	7.85 ± 0.46 ^b^	33.47 ± 1.11 ^b^
soybean	0.63 ± 0.01^d,e^	nd	1.45 ± 0.10 ^j,k^	14.69 ± 1.81 ^g^	5.41 ± 0.60 ^d^	22.18 ± 0.69 ^e^

Data are expressed as mean ± standard deviation of triplicate samples; Means in a column with different letters differ significantly (*p* < 0.05).

**Table 3:** Total phenolic content (TPC) and biological activities of beans.

	TPC	DPPH	α-Glucosidase inhibition(%)	BSA-Glucose (%)	BSA-MGO (%)	Tyrosinase inhibiton (%)
Lima bean	4.72 ± 0.23 ^c^	36.25 ± 1.02 ^f^	27.97 ± 1.07 ^g^	29.20 ± 0.41 ^l^	10.50 ± 0.78 ^h^	49.95 ± 1.32 ^e^
Broad bean	6.43 ± 0.71 ^b^	37.15 ± 2.14 ^e^	19.09 ± 1.05 ^i^	39.06 ± 1.95 ^f,g^	22.60 ± 1.02 ^f^	67.73 ± 1.28 ^c^
Common bean	8.59 ± 0.11 ^a^	46.83 ± 1.75 ^a^	51.74 ± 1.49 ^f^	86.67 ± 2.33 ^a^	74.06 ± 2.25 ^a^	75.89 ± 0.75 ^b^
Pea	4.87 ± 0.14 ^c^	31.92 ± 2.46 ^h^	16.17 ± 2.00 ^j^	36.04 ± 1.75 ^g,h^	21.65 ± 1.36 ^f^	38.62 ± 0.67 ^g^
Jack bean	3.77 ± 0.34 ^d,e^	37.81 ± 2.33 ^c,d^	32.53 ± 2.65 ^e,f^	55.66 ± 2.30 ^d^	16.92 ± 2.07 ^g^	48.11 ± 2.04 ^f^
Goa bean	2.44 ± 0.20 ^f,g^	37.15 ± 2.01^e^	60.42 ± 3.15 ^a^	40.18 ± 1.98 ^f,g^	3.74 ± 0.21 ^i^	23.05 ± 0.35 ^i^
Adzuki bean	2.68 ± 0.19 ^e,f^	18.08 ± 1.94 ^j^	64.33 ± 2.98 ^b^	47.32 ± 2.15 ^e^	12.70 ± 1.05 ^h^	38.92 ± 1.83 ^g^
Hyacinth bean	6.28 ± 0.23 ^b^	28.01 ± 1.17 ^i^	25.98 ± 3.01 ^h^	33.76 ± 2.69 ^h^	38.62 ± 2.46 ^d^	74.43 ± 1.25 ^b^
Chicking vetch	1.58 ± 0.14 ^g,h^	15.39 ± 1.48 ^k^	18.42 ± 1.33 ^i^	nd	nd	31.93 ± 0.78 ^h^
Garbanzo bean	1.04 ± 0.24 ^h^	1.28 ± 0.06 ^m^	15.90 ± 1.02 ^j^	nd	nd	21.35 ± 1.61 ^j^
Dral	7.95 ± 0.29 ^a^	37.93 ± 1.32 ^c^	32.14 ± 2.05 ^f^	68.16 ± 3.46 ^c^	62.46 ± 3.12 ^b^	67.96 ± 2.91^c^
Cow bean	3.94 ± 0.05 ^c,d^	37.27 ± 2.48 ^d,e^	51.54 ± 3.98 ^d^	42.66 ± 2.89 ^f^	29.56 ± 1.84 ^e^	47.49 ± 1.44 ^f^
Rice bean	4.88 ± 0.11 ^c^	35.36 ±1.99 ^g^	57.98 ± 4.47 ^c^	67.08 ± 3.75 ^c^	43.24 ± 1.36 ^c^	60.97 ± 0.19 ^d^
Mung bean	8.14 ± 0.21 ^a^	45.36 ± 1.27 ^b^	18.62 ± 2.84 ^i^	74.84 ± 4.08 ^b^	72.67 ± 2.57 ^a^	81.24 ± 1.51 ^a^
Soybean	6.10 ± 0.10 ^b^	15.17 ± 0.93 ^k^	12.06 ± 3.45 ^k^	58.50 ± 2.93 ^d^	17.44 ± 1.09 ^g^	39.02 ± 1.64 ^g^

nd means not detected; Data are expressed as mean ± standard deviation of triplicate samples; TPC was expressed as mg GAE/g; The anti-DPPH capacity was expressed as μM TE/g; Means in a column with different letters differ significantly (*p* < 0.05).

**Table 4:** Correlation coefficient of total phenolics acid, DPPH, α-glucosidase inhibition, BSA-MGO, BSA-Glucose and tyrosinase inhibition assay.

	TPC	DPPH	α-Glucosidase inhition	BSA-MGO	BSA-Glucose	Tyrosinase inhibition
total phenolic acids	0.671 a	0.578 a	0.322	0.676 a	0.812 b	0.639 a
TPC		0. 653 b	−0.123	0.609 a	0.848 b	0.883 b
DPPH			0.306	0.377	0.564 a	0.670 b
α-Glucosidase inhibition				0.148	−0.072	−0.018
BSA-MGO					0.787 b	0.494
BSA-Glucose						0.840 b

a Correlation is significant at *p* < 0.05 level (2-tailed);

b Correlation is significant at *p* < 0.01 level (2-tailed).
